# Prognostic Value of the Modified Systemic Inﬂammation Score in Patients With Extranodal Natural Killer/T-Cell Lymphoma

**DOI:** 10.3389/fphar.2020.593392

**Published:** 2020-09-30

**Authors:** He Huang, Li Min Chen, Xiao Jie Fang, Cheng Cheng Guo, Xiao Ping Lin, Huang Ming Hong, Xi Li, Zhao Wang, Ying Tian, Mei Ting Chen, Yu Yi Yao, Zegeng Chen, Xiao Qian Li, Fei Pan

**Affiliations:** ^1^ Department of Medical Oncology, Sun Yat-sen University Cancer Center (SYSUCC), Guangzhou, China; ^2^ State Key Laboratory of Oncology in South China, Sun Yat-sen University Cancer Center (SYSUCC), Guangzhou, China; ^3^ Collaborative Innovation Center for Cancer Medicine, Sun Yat-Sen University Cancer Center, Guangzhou, China; ^4^ Department of Neurosurgery, Sun Yat-sen University Cancer Center, Guangzhou, China; ^5^ Department of Nuclear Medicine, Sun Yat-sen University Cancer Center, Guangzhou, China; ^6^ Department of Medical Oncology, Sun Yat-sen Memorial Hospital, Guangzhou, China

**Keywords:** extranodal natural killer/T cell lymphoma, systemic inﬂammation score, neutrophil-lymphocyte ratio, albumin, prognosis

## Abstract

**Background:**

Extranodal natural killer/T-cell lymphoma (ENKTL) is a rare and extremely malignant tumor. The systemic inﬂammation score (SIS), which is based on the pretreatment level of lymphocyte-to-monocyte ratio (LMR) and serum albumin (Alb), has been shown to be of prognostic value in a number of cancers. We integrate several other pretreatment serum inflammatory indicators, including the neutrophil-to-lymphocyte ratio (NLR), lymphocyte-to-monocyte ratio (LMR), serum C-reactive protein (CRP) and albumin (Alb) level, to establish a modified systemic inflammatory scoring system to predict clinical outcomes of ENKTL.

**Methods:**

A total of 184 patients with newly diagnosed ENKTL was retrospectively investigated. Systemic inflammatory indexes, including NLR, LMR, CRP, and Alb level were reviewed. Receiver operating characteristic (ROC) curve analysis was carried out to obtain the optimal cut-off value. The associations between cutoff values and overall survival (OS) were analyzed by Kaplan–Meier curves and Cox proportional models.

**Results:**

The median age of patients was 44.0 years, ranging from 15 to 82 years. There were 129 (70.1%) male patient. About 57.1% of patients had stage III or IV disease. The optimal cut-off values of NLR and LMR in predicting OS were 3.1 and 2.4, respectively. The clinical standard of CRP and Alb levels at 10 and 40 mg/L, respectively, were chosen as the optimal cut-off values. By multivariate analysis, hemophilic syndrome (hazard ratio [HR]: 10.540, 95% confidence interval [CI]: 3.440–32.291, P < 0.001), advanced Ann Arbor stages (III–IV) (HR: 4.606, 95% CI: 1.661–12.774, P = 0.003), paranasal sinus invasion (HR: 2.323, 95% CI: 1.069–5.047, P = 0.033), NLR ≥ 3.1 (HR: 3.019, 95% CI: 1.317–6.923, P = 0.009), Alb level of <40 mg/L (HR: 0.350, 95% CI: 0.134–0.915, P = 0.032), and radiation therapy (HR: 0.430, 95% CI: 0.205–0.901, P = 0.025) were independent protective factors for ENKTL. We combined two inflammatory indexes NLR and Alb level to establish a modified systemic inﬂammation score (mSIS). These 184 patients were divided into 3 groups: group 1 (mSIS score of 0), group 2 (mSIS score of 1), and group 3 (mSIS score of 2). The mean OS of these three groups were 42 months (95% CI: 31.4–53.12), 77 months (95% CI: 68.5–87.5), and 89 months (95% CI: 71.4–82.7), respectively (P < 0.001). The Harrell’s concordance index (C-index) of mSIS is 0.725. The mSIS could be used to discriminate patients categorized in the low-risk group of International Prognostic Index (IPI) (P < 0.001) and the low-risk and intermediate-risk prognostic index of natural killer cell lymphoma (PINK) group (P = 0.019).

**Conclusion:**

The pretreatment mSIS could be an independent prognostic factor for OS in patients with ENKTL and warrants further research.

## Introduction

Extranodal natural killer/T-cell lymphoma (ENKTL) is a rare and highly malignant lymphoma with heterogeneous clinical behaviors. Most of the tumors occur primarily in the nose and above the throat, and the malignant cells of ENKTL mainly derived from mature NK cells, or T cells, therefore it is named NK/T cell lymphoma (Kwong, 2005; Freud et al., 2006; Sun and Lanier, 2011; Tse and Kwong, 2013; Montaldo et al., 2014). A few cases may occur outside of the nose, such as skin, gastrointestinal tract, lung, etc. Its pathological manifestations are unique, with vascular center of pleomorphic lymphocyte infiltration. The tumor cells infiltrate to destroy the blood vessels and then cause tissue necrosis (Kwong and Khong, 2011). In most cases, there is evidence of Estein-Barr virus infection (Rickinson et al., 1992).

Systemic inflammation has been shown to play a considerable role in the progression and development of cancers (McMillan et al., 2003). NK/T lymphoma is associated with inflammatory cytokine storm, leading to complications such as hemophagocytic syndrome in later stage.

Potential prognostic values have been shown by pretreatment serum inﬂammatory indicators like the neutrophil-to-lymphocyte ratio (NLR), lymphocyte-to-monocyte ratio (LMR), the C-reactive protein (CRP), and albumin (Alb) levels in some neoplasms (McMillan, 2013; Kim et al., 2015; Akinci Ozyurek et al., 2017; Lin et al., 2017; Rajwa et al., 2018). However, there is no generally accepted optimal threshold for inﬂammatory biomarkers and no scoring system to incorporate these indicators in ENKTL. This study aims to look through the prognostic value of some pretreatment serum-based inﬂammatory biomarkers to establish a scoring system for the prognosis of patients with ENKTL.

## Patients and Methods

### Ethical Statement

This study was approved by the Medical Ethics Committees of Sun Yat-sen University Cancer Center. The study procedures were conducted in accordance with the World Medical Association Declaration of Helsinki. Written informed consents were provided by patients to use data stored in the hospital database.

### Study Population

The database of 204 patients with diagnosed ENKTL from December 28th 2010 to May 1st 2019 in Sun Yat-sen University Cancer Center was surveyed retrospectively. The inclusion criteria include: (1) pathologically diagnosed ENKTL according to the WHO classification of tumors of hematopoietic and lymphoid tissues; (2) no preceding malignant or secondary tumor; (3) no preceding treatment; (4) the subsequent treatment was chemotherapy with or without radiation or radiotherapy alone to achieve the therapeutic intent; (5) complete followed up data and clinical information. The exclusion criteria were: (1) neoadjuvant chemotherapy, (2) no routine blood examination before treatment, (3) incomplete/inaccurate medical records, and (4) acute chronic active inflammatory or infectious diseases.

### Data Collection

The following baseline clinical information were collected by medical record: age, gender; Karnofsky performance status (KPS); Ann Arbor stage; B symptoms; pathological diagnosis; EBV infection status; computed tomography (CT) or magnetic resonance (MR) image of the neck, nasopharynx, abdomen, chest, and pelvis; or positron discharge tomography/computed tomography (PET/CT) of the entire body. All the patients were evaluated with blood cell counts, serum CRP, and Alb levels before treatment and were followed up from the date of diagnosis. They were followed up generally every 3 months in the first year, every 6 months in the second and third year, and every year thereafter. At the last follow-up date, clinical attendance was made by direct communication with the patient or his or her family. Overall survival (OS) was counted from the date of diagnosis to the date of death or the final follow-up.

### Statistical Analysis

We apply Chi-square test or Fisher’s exact test to evaluate varying baseline and clinicopathological parameters according to the variety of data. The optimal cut-off value for the systemic inflammatory indexes NLR and LMR was determined by the receiver-operating-characteristic (ROC) curve. The optimal cut-off value of Alb and CRP are their clinical standard values. The log-rank test was applied to analyze the difference between the curves. Univariate or multivariate death hazard ratios(HRs) were calculated by Cox proportional hazard model. All reported P-values were two-sided, and P < 0.05 was considered to be statistically significant.CIs were calculated at the 95% level. The survival curves were figured by Kaplan-Meier survival analysis. Discrimination was measured by Harrell’s concordance index (C-index), which quantifies the likelihood of two random patients. The patient who relapses for the first time had a higher possibility of interest event.

The ROC curve was executed using the MedCalc Statistical Software version 18.2.1 (MedCalc Software bvba, Ostend, Belgium).The C-index was calculated by R version 4.0.2 *via* the survival and design packages, while other analyses were performed using the SPSS software version 25.0 (IBM Corp, Armonk, NY, United States).

## Results

### Patient Characteristics

According to the including and excluding criteria above, 20 patients were excluded. Therefore, 184 patients were enrolled and analyzed in this study. The baseline characteristics of these patients were showed in **Table 1**. The median age was 44.0 years, ranging from 15 to 82 years. The age interquartile range (IQR) was 34.0–54.0 years. There were 129 (70.1%) male and 55 (29.9%) female patients. A total of 146 (79.3%) patients had KPS higher than 80.A total of 113 patients (61.4%) had B symptoms at the initial assess, 57.1% of patients had stage III or IV disease, and 164 (89.1%) of patients had nasal type ENKTL. Thirty-four patients had more than one extranodal involvement. Some patients had metastases to the liver (one case), lung (two case), kidney (one case), adrenal gland (three cases), skin (two cases), and gastrointestinal tract (two cases). The presence of EBV DNA was measured in the plasma or whole blood at diagnosis (n = 124). Plasma lactate dehydrogenase(LDH) was found to be elevated in 33 cases (17.9%). According to the International Prognostic Indexs (IPI) (International Non-Hodgkin’s Lymphoma Prognostic Factors Project, 1993), the majority of the patients (133 cases, 72.3%) were categorized as low risk (IPI = 0–1) and other patients (51cases, 27.7%) as low-intermediate/intermediate-high/high risk (IPI = 2−5). The patients with Korean Prognostic Indexs (Lee et al., 2006) (KPI) = 0−1 (117 cases, 63.6%) was significantly more than those with KPI = 2−4(67 cases, 36.4%). For the prognostic index of natural killer cell lymphoma (PINK) index (Kim et al., 2016), 63 cases (34.2%) were classified as low risk, 42 cases (22.8%) as intermediate risk, and 79 cases (42.9%) as high risk. There are 110 cases (59.8%) of patients had received radiation therapy.

**Table 1:** Baseline characteristics of patients.

Characteristics	No. of patients	%
**Age [median (range), years]**		
<60	153	83.2
≥60	31	16.8
**Gender**		
Male	129	29.9
Female	55	70.1
**KPS score**		
≤80	38	20.7
>80	146	79.3
**Ann Arbor stages**		
I–II	79	42.9
III–IV	105	57.1
**B symptoms**	113	61.4
**Hemophilic syndrome**	9	4.9
**Bone marrow involvement**	16	8.7
**Subtypes**		
Nasal	164	89.1
Non-nasal	20	10.9
**Extranodal involvement sites**		
<2	150	81.5
≥2	34	18.5
**Lymph-node involvement**	115	62.5
**Plasma Epstein-Barr virus DNA(+)**	124	67.4
**LDH elevated**	33	17.9
**Paranasalsinus invasion**	77.2	22.8
**Korean Prognostic Indexs (KPI) score**		
0–1	117	63.6
2–4	67	36.4
**International Prognostic Indexs(IPI) score**		
0–1	133	72.3
2–5	51	27.7
**The prognostic index of natural killer lymphoma (PINK) model**		
Low-risk	63	34.2
Intermediate-risk	42	22.8
High-risk	79	42.9
**Radiation therapy**	110	59.8

### Optimal Cut-Off Values of Systemic Inflammatory Indexes

**Figure 1:**
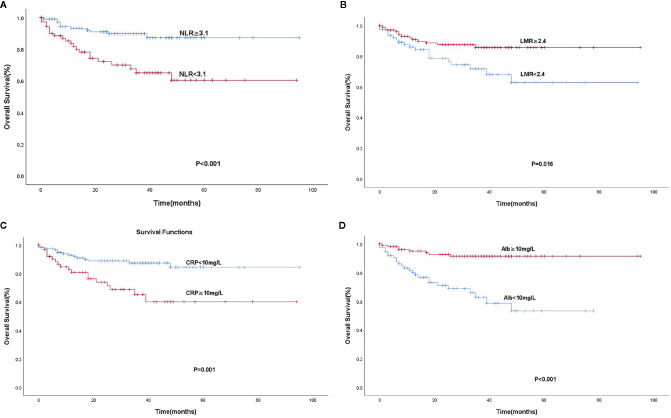
Kaplan-Meier analysis for OS according to the Optimal cut-off values of NLR **(A)**, LMR **(B)**, CRP **(C)**, and Alb **(D)**.

According to the ROC curve, the area under curve (AUC) of NLR was 0.669 (95% C: 0.596–0.735; P < 0.05) based on a cut-off value of 3.1. Similarly, the AUC of LMR was 0.623 (95% CI: 0.548–0.693; P < 0.05) based on a cut-off value of 2.4. As mentioned above, the optimal cut-off values of levels of CRP and Alb were 10 and 40 mg/L, respectively. The NLR-low group (60.9%, 112/184) had a higher OS than the NLR-high group (39.1%, 72/184; P < 0.001). The OS was significantly poorer in the LMR-high group (57.6%, 106/184) than in the LMR-low group (42.4%, 78/184; P = 0.015). The CRP-low group (64.1%, 118/184) had a higher OS than the CRP-high group (35.9%, 66/184; P = 0.001), and the OS was significantly shorter in the Alb-low group (41.8%, 77/184) than in the Alb-high group (58.2%, 107/184; P < 0.001) (**Figure 1**).

### Association of Systemic Inflammatory Indexes With OS

We evaluated the association of systemic inflammatory indexes with the OS of patients. The median follow-up period was 28.5 months. The 5-year OS rates for the whole cohort were 60.2%. The univariate and multivariate survival analyses are presented in **Table 2**. In the univariate analysis, the following factors significantly predicted poor outcome:low KPS score (KPS ≤ 80),B symptoms, advanced Ann Arbor stage (III/IV), hemophilic syndrome, bone marrow involvement, regional lymph node involvement, paranasal sinus invasion, positive plasma EBV-DNA, CRP of ≥10mg/L, Alb level of <40 mg/L, NLR ≥ 3.1, LMR < 2.4, and IPI score≥2. The multivariate analysis was performed on the clinical parameters related to shorter overall survival and important clinical factors, such as age and serum LDH level. We used conditional Cox regression analysis and found four negative prognostic factors on OS:Hemophilic syndrome (HR: 10.540, 95% CI: 3.440–32.291; P < 0.001), advanced Ann Arbor stages (III–IV) (HR: 4.606, 95% CI: 1.661–12.774; P = 0.003), paranasalsinus invasion (HR: 2.323, 95% CI: 1.069–5.047;P = 0.033), NLR ≥ 3.1 (HR: 3.019, 95% CI: 1.317–6.923; P = 0.009), Alb level of <40mg/L (HR: 0.350, 95% CI: 0.134–0.915; P = 0.032), and radiation therapy (HR: 0.430, 95% CI: 0.205–0.901; P = 0.025).

**Table 2:** Univariate and multivariate analyses of potential prognostic factors for OS.

Characteristics	Univariate analysis		Multivariate analysis	
	HR (95% CI)	P	HR (95% CI)	P
**Age**				
<60				
≥60	2.060 (0.953–4.453)	0.066		0.51
**Gender**				
Male				
Female	1.190 (0.550–2.574)	0.658****		
**KPS score**				
≤80				
>80	0.426 (0.201–0.904)	0.026		0.297
**Ann Arbor stages**				
I–II				
III–IV	5.675 (2.177–14.796)	0	4.606(1.661–12.774)	0.003
**B symptoms**	3.154 (1.297–7.673)	0.011		0.341
**Hemophilic syndrome**	8.098 (3.290–19.929)	0	10.540(3.440–32.291)	0
**Bone marrow involvement**	4.390 (1.886–10.215)	0		0.891
**Subtypes**				
Nasal				
Non-nasal	1.071 (0.325–3.527)	0.91		
**Extranodal involvement sites**				
<2				
≥2	2.057 (.918–4.611)	0.08		
**Lymph-node involvement**	4.301 (2.067–8.950)	0		0.354
**Epstein-Barr virus infection**	3.357 (1.289–8.741)	0.013		0.312
**Paranasalsinus invasion**	2.303 (1.107–4.789)	0.026	2.323(1.069–5.047)	0.033
**CRP ≥ 10mg/l**	2.964 (1.470–5.975)	0.002		0.056
**NLR ≥ 3.1**	3.448 (1.661–7.157)	0.001	3.019(1.317–6.923)	0.009
**LMR ≥ 2.4**	0.428 (0.211–0.867)	0.018		0.791
**ALB < 40 mg/L**	5.531 (2.396–11.949)	0	0.350(0.134–0.915)	0.032
**LDH elevated**	2.112(0.943–4.727)	0.069		
**KPI score ≥ 2**	1.838 (0.910–3.711)	0.09		
**IPI score ≥ 2**	2.365 (1.144–4.889)	0.02		0.734
**Radiation therapy**	0.353(0.175–0.713)	0.004	0.430(0.205–0.901)	0.025

### A Modified Systemic Inﬂammation Score

In previous studies, we found that the systemic inﬂammation score (SIS), which is based on the preoperative Alb level and LMR, was a prognostic factor in renal clear cell carcinoma and colorectal cancer. Based on the above results in this study, LMR could not predict survival in multivariate analysis. Intriguingly, high NLR and low level of Alb had poor overall survival.

Whether the combination of Alb level and NLR could predict the prognosis of patients with ENKTL remained unclear. Here, we established a modified systemic inﬂammation score (mSIS) to demonstrate the prognostic value of the combination of Alb level and NLR for the prognosis of ENKTL patients.

**Figure 2:**
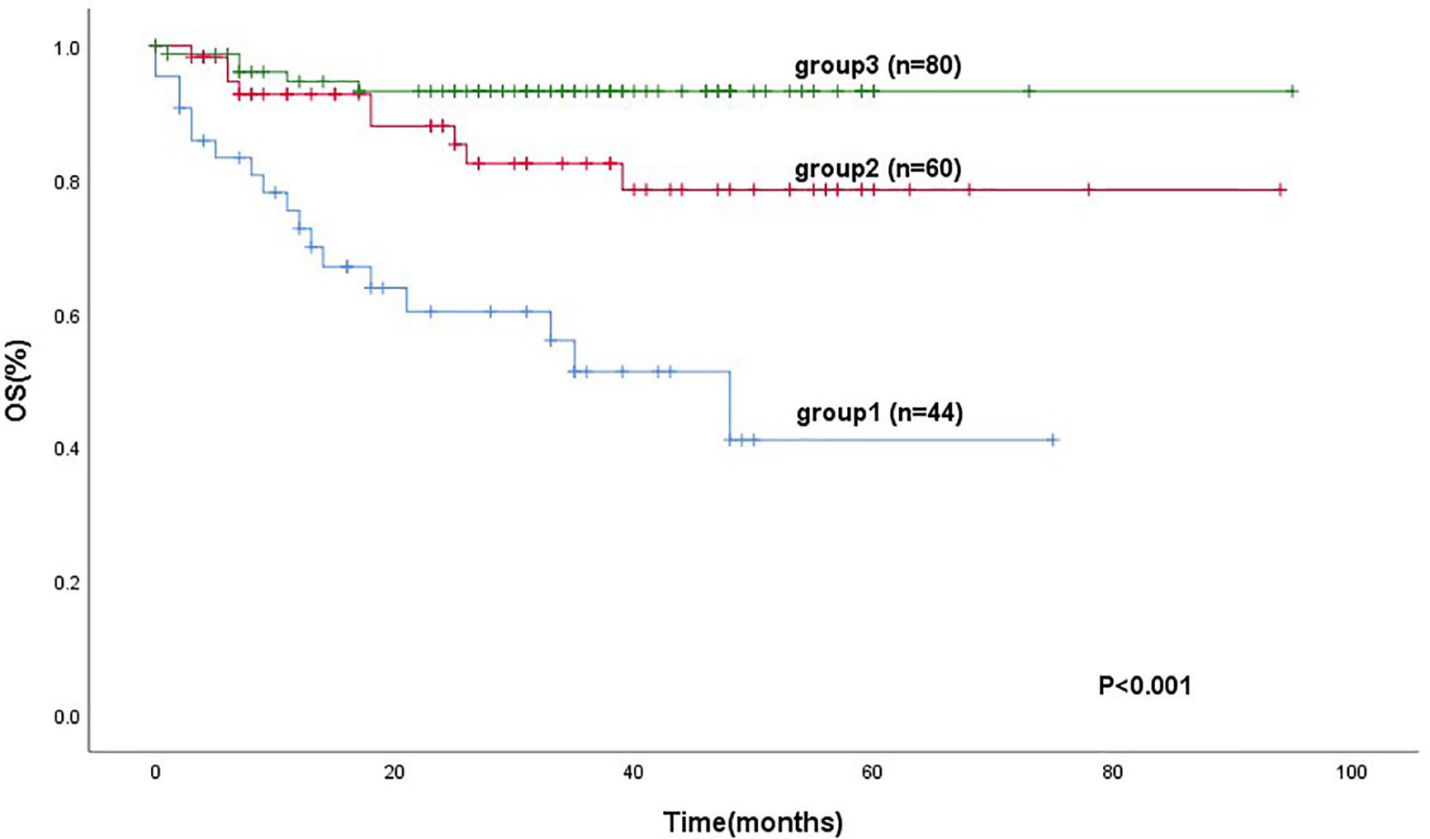
Kaplan-Meier analysis for OS according to the mSIS.

184 patients were divided into three groups. Group 1 (mSIS score of 0), patients with NLR ≥ 3.1 and Alb level of <40 g/L (44 cases, 23.9%). Group 2 (mSIS score of 1), patients with NLR < 3.1 and Alb level of <40 g/L or patients with NLR ≥ 3.1 and Alb level of ≥40 g/L (60 cases, 32.6%). Group 3 (mSIS score of 2), patients with NLR < 3.1 and Alb level of ≥40 g/L (80 cases, 43.5%). The mean for OS of these three groups were 42 months (95% CI: 31.4–53.12), 77 months (95% CI: 68.5–87.5), 89 months (95% CI: 71.4–82.7), respectively (P < 0.001; **Figure 2**). The C-index of mSIS is 0.725.

**Figure 3:**
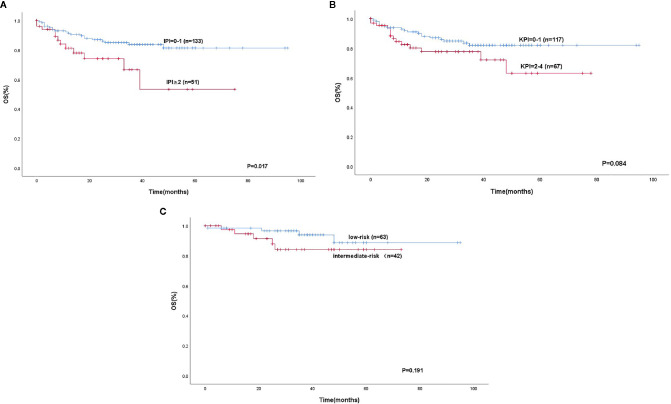
Kaplan-Meier analysis for OS according to the IPI **(A)**, KPI **(B)**, low-risk and intermediate-risk according to the PINK **(C)**.

**Figure 4:**
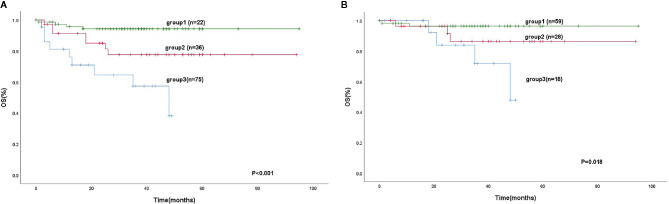
Kaplan-Meier analysis for OS according to the IPI (0-1) **(A)**, low-risk and intermediate-risk according to the PINK **(B)**, as determined by the mSIS.

We separated our patients according to the IPI, KPI, and PINK. The patients could be stratified into two risk groups based on IPI (IPI, 0-1 vs. ≥2, P < 0. 0 0 1), 133 (72.3%) patients were in the low-risk group, and 51 patients were in the high-risk group. Using the KPI, patients were unable to be distinguished in two risk groups (P = 0.084). Using the PINK, there was no difference in the survival difference between the low-risk and intermediate-risk groups (P = 0.199) (**Figure 3**). However, the mSIS can classify the patients into the low-risk group of the IPI (P < 0.001) and the low-risk and intermediate-risk PINK group (P = 0.019; **Figure 4**).

## Discussion

ENKTL is usually associated with poor prognosis. Tremendous efforts have been made in searching valid survival indicators for patients with ENKTL. Numerous studies have demonstrated that cancer progression is caused by the intrinsic properties of tumor cells as well as the systemic and local inflammatory responses. Cancer can cause local and systemic inflammation, which is thought to be associated with cancer and is a hallmark of cancer development and progression (Rajendran et al., 2018). Different systemic inflammatory indicators including NLR, LMR, CRP, and Alb have been analyzed in several malignant tumors with poor outcome and low therapeutic response (Lee et al., 2006; Stotz et al., 2014; Gu et al., 2015; Koh et al., 2015; Kim et al., 2016; Jing et al., 2017; Rajendran et al., 2018). The optimal cut-off values for these systemic inflammatory biomarkers are distinct in a large number of malignancies (Stotz et al., 2014; Chen et al., 2015; Gu et al., 2015; Kim et al., 2015; Koh et al., 2015; Kim et al., 2016; Jing et al., 2017; Tan et al., 2017). We investigated their prognostic significance in ENKTL in this study.

In order to confirm the optimal cutoff value of those indexes in predicting the prognosis of ENKTL, we used a ROC curve analysis. According to the ROC curve and the Youden index, the optimal cut-off values of NLR,LMR in predicting OS were 3.1 and 1.4, respectively. Based on these value, 184 patients were divided into low- and high-risk groups. Based on the results, NLR (P = 0.009) and Alb level (P = 0.032) were shown to be able to predict the OS in patients with ENKTL. At the same time, hemophilic syndrome (P < 0.001), paranasalsinus invasion (P = 0.033), advanced Ann Arbor stages (III–IV) (P = 0.001), and radiation therapy (P = 0.025) also considerably predicted poor survival in multivariate analysis.

The systemic inﬂammation score (SIS), which is based on the pretreatment level of lymphocyte-to-monocyte ratio (LMR) and serum albumin (Alb). In the current study, we creatively establish a modified systemic inﬂammation score (mSIS) as a novel, straightforward, and valuable prognostic indicator for OS in for patients with ENKTL, which is based on more than preoperative NLR and serum Alb level.

The mSIS on prognosis of ENKTL is constructed primarily by the NLR and Alb level. Neutrophils and lymphocytes form NLR. Neutrophils can produce chemokines and cytokines to inhibit the immune activity of lymphocytes and natural killer cells. The interaction between neutrophils and cancer cells could produce inflammatory response, which cause the proliferation, invasion and metastasis of tumors (Petrie et al., 1985). On the contrary, lymphocytes, the basic component of adoptive immune system (Dunn et al., 2004), could enhance tumor immune monitoring and suppress the proliferation, invasion, and metastasis of tumor cells. In our study, NLR ≥ 3.1 significantly predicted poor survival in univariate analysis Therefore, NLR is important in the prognosis of ENKTL. The level of Alb, which is widely applied as an excellent indicator of malnutrition and cachexia in patients with advanced cancer. It has been found that Alb of <40 mg/L was remarkably predict shorter overall survival in univariate analysis. Therefore, the mSIS may enable to assess the impact of tumors on systemic inflammation and malnutrition.

However, although the prognostic value of mSIS has been shown for predicting the survival of patients with ENKTL, the mechanism is still unclear. A lower score of mSIS indicates an elevated NLR and/or low level of albumin, which might indicate immunological response, malnutrition, and cachexia.

On one hand, neutrophils secrete reactive oxygen species that could lead to cellular genetic instability and DNA damage and promote tumor microenvironment to induce carcinogenesis (Weitzman et al., 1990). Neutrophils can also secrete vascular endothelial growth factor (VEGF), which promote tumor cell cycle by activating VEGF receptor 2 (Shamamian et al., 2001; Kusumanto et al., 2003). Lymphocytes are a basal anti-tumor defense line, a reduced number of lymphocytes is associated with unstable host defense to the advanced tumors. There are some subsets of lymphocytes including CD4+ T helper type 1 lymphocytes,CD8+ cytotoxic T lymphocytes and natural killer T cells. They were shown to have anticancer abilities detecting precancerous cells and destroying them, directly killing cancer cells, and preventing angiogenesis and tumor metastasis (Lowther and Hafler, 2012; Li et al., 2014). Moreover, neutrophils have been reported to inhibit T cell activation by producing nitric oxide, arginase, and reactive oxygen species, bringing about the depletion of the lymphocyte immune response (Muller et al., 2009). These may explain the reason that elevated NLR may serve as an independent prognostic factor for OS owing to the counts of increased neutrophils and reduced lymphocytes.

On the other hand, albumin is considered as a nutritional indicator. In recent studies, albumin has become a common indicator for predicting survival of a variety of cancers, such as osteosarcoma, renal cell carcinoma colorectal cancer, hepatocellular carcinoma, and prostate cancer (Yi et al., 2014; Chen et al., 2015; Nazha et al., 2015; Chi et al., 2016; Hiraoka et al., 2016). The mechanisms might involve in malnutrition and tumor microenvironmental imbalance. High concentrations of interleukin 6 (IL-6) and tumor necrosis factor (TNF) regulate liver cells to generate albumin production and increase the permeability of the microvasculature. It has been validated that albumin can maintain cell growth and DNA replication, and produce antioxidant effects on carcinogens (Arroyo et al., 2014). In addition, malnutrition will weaken the human body’s cellular immunity,h umoral immunity, phagocytosis, and other defense mechanisms, thus leading to an increased possibility of infection and poor anti-tumor treatment effect (Chandra et al., 1999).

This study suggested that mSIS is superior to IPI,KPI and PINK, and could be a new prognostic scoring system model contributes to the risk assessment for patients with ENKTL.

There are some limitation in our study. Firstly, as a retrospective study with a moderate sample size and a single center design, patient selection bias may exist. Secondly, it is difficult to keep the heterogeneity in the treatment used for each patient, which result in different clinical prognosis.Nevertheless, prospective trials are warranted for future study.

In conclusion, our study indicates that mSIS can disclosed the systemic inflammatory reaction and damaged nutritional status and therefore can forecast the survival of patients with ENKTL. Patients with higher mSIS had better OS. mSIS will help direct individual treatment for patients with ENKTL. Patients with low mSIS should be well monitored and treated more seriously to avoid tumor progression.

## Data Availability Statement

The raw data supporting the conclusions of this article will be made available by the authors, without undue reservation, to any qualified researcher.

## Ethics Statement

The studies involving human participants were reviewed and approved by ClinicalTrials.gov. Written informed consent to participate in this study was provided by the participants’ legal guardian/next of kin. Written informed consent was obtained from the individual(s) for the publication of any potentially identifiable images or data included in this article.

## Author Contributions

These authors contributed equally: HH, LC, and XF. All authors contributed to the article and approved the submitted version.

## Funding

This work was supported by Science and Technology Planning Project of Guangdong Province, China (grant number 2017A020215030), Basic and Applied Basic Research Fund Project of Guangdong Province, China (grant numbers 2019A1515010742 and 2019A1515010702]. The funders had no role in the study design, data collection and analysis, decision to publish, or preparation of the manuscript.

## Conflict of Interest

The authors declare that the research was conducted in the absence of any commercial or financial relationships that could be construed as a potential conflict of interest.
